# The value of predictive models assisted diagnosis of benign and malignant lacrimal gland tumor based on dynamic contrast-enhanced combined with conventional MRI

**DOI:** 10.1515/med-2026-1437

**Published:** 2026-06-15

**Authors:** Tianming Jian, Fei Gao, Mei Yang, Tong Wu, Xiaodong Ji, Shuang Xia, Fengyuan Sun

**Affiliations:** Tianjin Key Laboratory of Retinal Functions and Diseases, Tianjin Branch of National Clinical Research Center for Ocular Disease, Eye Institute and School of Optometry, Tianjin Medical University Eye Hospital, Tianjin, China; Department of Ophthalmology, Taizhou Hospital of Zhejiang Province Affiliated to Wenzhou Medical University, Taizhou, China; Department of Orbital Disease and Oculoplastic Surgery, Sichuan Eye Hospital, Aier Eye Hospital Group, Chengdu, China; Department of Radiology, Tianjin First Central Hospital, Tianjin, China

**Keywords:** MRI, dynamic contrast enhancement, quantitative parameter, lacrimal gland tumor, predictive model

## Abstract

**Background or Purpose:**

To explore the value of predictive models assisted diagnosis of benign and malignant lacrimal gland tumor based on dynamic contrast enhanced (DCE) combined with conventional MRI.

**Methods:**

This study retrospectively analyzed the patients with primary lacrimal gland tumors using conventional and DCE-MRI. 15 base predictive models were conducted to compare the diagnostic value.

**Results:**

A total number of 76 patients were enrolled, including 41 were malignant, 35 were benign. Compared with the benign and malignant tumors, MRI parameters including tumor diameter, tumor shape, adjacent bone damage, K^trans^, K_ep_, V_e_ and TIC were selected into predictive models. Six-performance metrics of models with DCE quantitative parameters was significantly better than the models without. According to the test results, Extra Trees Classifier (ET) showed the highest MCC and F1 score, the accuracy was 91 %, precision was 100 %, and the recall was 83 %, based on DCE quantitative parameters plus conventional MRI features.

**Discussion or Conclusions:**

Our findings indicate that the predictive models based on DCE quantitative parameters plus conventional MRI features have shown better performance than the models based on conventional MRI features alone. ET could be selected as the best model in the diagnosis of benign and malignant lacrimal tumors.

## Introduction

The lacrimal gland tumors account for approximately 5–13 % of all biopsied orbital masses, encompassing both benign and malignant tumors originating from the epithelium and non-epithelium [1], 2]. Considerable differences exist between the biological behavior, therapy, and prognosis of benign and malignant diseases. Benign epithelial tumors, such as pleomorphic adenomas, exhibit expansive growth patterns and demonstrate a low recurrence rate following complete resection [3]. Conversely, malignant epithelial tumors, such as adenoid cystic carcinoma, are characterized by invasive growth, should be administered with extensive resection, adjuvant radiotherapy and chemotherapy to reduce the risk of local recurrence [4], 5]. For non-epithelial lesions, such as lymphoid hyperplasia or lymphoma, the selection of corticosteroid therapy or systemic chemotherapy should be based on the specific pathological subtype [6]. Therefore, preoperative diagnosis of benign and malignant diseases by distinguishing imaging characteristics of the lesions is an extremely challenging and crucial task. MRI plays a vital role in the preoperative diagnosis of orbital tumors, owing to its superior ability in displaying tumor morphology, texture, and invasion range. However, these imaging assessment methods are often subjective, and conventional MRI lacks sufficient sensitivity to distinguish the non-specific or early-stage malignant tumors from the benign ones [7], 8].

Dynamic contrast-enhanced MRI (DCE-MRI) employs perfusion imaging techniques to generate time-intensity curve (TIC) of contrast agent in tissues, has been used in the diagnostic assessment of a range of tumors [9], [10], [11]. The Tofts pharmacokinetic model is widely used to obtain quantitative analysis, and the parameters including forward volume transfer constant (K^trans^), reverse transfer rate constant (K_ep_), plasma volume fraction (V_p_), and the interstitial volume fraction (V_e_) [12], [13], [14]. These features are known to be related to cancer driven angiogenesis, incomplete vascular structure and overly permeability [15], thereby enabling the differentiation between benign and malignant orbital tumors [16], [17], [18]. Despite the potential of DCE quantitative parameters in characterizing tumor vascularity, prior studies have shown limited diagnostic efficacy when these parameters are used in isolation, but they may have a role in adjunctive imaging [19]. Recently, two studies demonstrated the effective utilization of DCE and diffusion-weighted imaging MRI in differentiating between benign and malignant lacrimal tumors [20], 21]. Our previous research demonstrated the predictive value for the diagnosis of benign and malignant epithelial tumors by combination DCE quantitative parameters and conventional MRI features [22]. Currently, literature specific to DCE and lacrimal gland malignancies remains limited, and it is difficult to explain the complex relationships among the DCE parameters by conventional statistics. Machine learning encompassing specific algorithms that facilitate capturing nonlinear relationships and high-order interactions, can be used in further validation. However, no study has been conducted to screen predictive models for assisting in the diagnosis of lacrimal gland tumors by machine learning.

Therefore, in this study, we intended to explore the potential of predictive models for assessing tumor malignancy in lacrimal gland tumors by combining DCE quantitative parameters with conventional MRI features.

## Methods

### Study design and patients

Patient data of lacrimal gland tumors were collected at our Hospital from January 2015 to December 2021. The following inclusion criteria were applied: (1) All patients underwent both conventional MRI and DCE-MRI; (2) The pathological diagnosis was confirmed. The exclusion criteria were as follows: (1) Previous biopsy, surgery, radiation, or chemotherapy; (2) Secondary, or metastatic tumor. Finally, a total of 76 patients were included in the study. Demographic data such as age, gender, laterality, medical history, and histopathology were extracted from medical records.

### MRI protocol

Imaging was performed using a 3 Tesla scanner (Magnetom Trio Tim; Siemens Healthcare, Erlangen, Germany) with an 8-channel headcoil. The parameters were as follows: T1-weighted imaging (T1WI): repetition time (TR) 500 ms, echo time (TE) 8.2 ms, section thickness (ST) 2.5 mm, matrix 260 × 213; T2-weighted imaging (T2WI): TR 6000 ms, TE 94 ms, ST 2.5 mm, matrix 320 × 288. DCE-MRI was acquired by using 3D time-resolved imaging with interleaved stochastic trajectory (3D-TWIST) sequences. The scanning parameters: TR 5 ms, TE 2 ms, ST 3 mm, intersection gap 3 mm, flip angle 12°, matrix 320 × 189, field of view 170 × 240 mm. The contrast agent, gadopentetic acid dimeglumine salt (Magnevist; Bayer Schering, Berlin, Germany), was administered intravenously at an injection flow rate of 2.5 mL/s (total dose, 0.1 mmol/kg of body weight), followed by a 20-mL saline solution flush applied at the same rate.

DCE-MRI data were processed using a dedicated postprocessing software program (Tissue 4D; Siemens Healthcare). The 2 × 2 mm^2^ region of interest (ROI) was drawn manually on the tumor’s solid area showing the greatest degree of early enhancement on DCE-MRI, avoiding the areas of vessel, cyst, necrosis, and margin.

### MRI data collection

Conventional MRI features, such as tumor’s maximum diameter, tumor shape, adjacent bone damage, internal signal intensity, enhancement homogeneity, and cystic change were collected. The maximum diameter of the mass was determined on T1WI or T2WI transverse planes. Based on the characteristics of growth, the tumor shape in the transverse section were previously divided into three categories: (1) Oval; (2) Wedge sign (infiltration into the posterior orbit can form a clear lateral triangle of tissue); and (3) irregular or indistinguishable shape [23], 24]. Adjacent bone damage were divided into two categories: (1) without damage (no change and bone remodeling present); (2) with damage (bone erosion/destruction, showing the continuity of the hypointense contour in the cortical bone interrupted by local isointense) [25]. The tumor’s signal intensity in T1WI and T2WI was compared with that of ipsilateral extraocular muscle, which was divided into hypointense, isointense, and hyperintense.

DCE-MRI parameters, such as TIC, K^trans^, K_ep_, and V_e_ were documented. The TIC was divided into three types: (I) Persistent type: the signal intensity showing continuous enhancement during the dynamic observation time; (II) Plateau type: showing a relatively prominent increase slope and reaching a final intensity 90–100 % of the peak signal intensity; (III) Washout type: displaying a rapid increase slope followed by a final intensity lower than 90 % of the peak signal intensity [17]. A modified Tofts model was used to calculate the pharmacokinetic parameters, including: (1) K^trans^, volume transfer constant between blood plasma and extravascular extracellular space in abnormal tissue; (2) K_ep_, reverse volume transfer constant/efflux rate constant from extravascular extracellular space to blood plasma; (3) V_e_, volume fraction of the extravascular extracellular space volume per unit volume of tissue [15].

The measurements were independently conducted by two head and neck radiologists (S. Xia and X. Ji) who were blinded to the clinical and histopathologic results. All measurements were repeated by a reader (S. Xia), spaced at least one month apart. The average of the two measurements was subsequently subjected to statistical analysis.

### Statistical analysis

The Shapiro-Wilk test was used for the assessment of normality. Continuous variables were expressed as mean ± SD or M (Q_1_, Q_3_) based on the results of the normality tests. Categorical variables were expressed as number (%). Intra-observer and inter-observer reproducibilities were assessed via intraclass correlation coefficient (ICC), interpreted as follows: <0.4, poor agreement; ≥0.4 and <0.6, moderate agreement; ≥0.6 and <0.8, good agreement; and ≥0.8 excellent agreement. The differences between benign and malignant groups were compared by using Student’s *t-*test, Mann-Whitney *U*‐test, *χ* ^*2*^ *-*test, or Fisher exact-test, where appropriate. The variables at a significance level of p<0.15 were then entered into predicative models. The data were analyzed by using SPSS 25.0 (NY, USA). p<0.05 was considered to indicate a statistically significant difference.

The 76 samples were divided into training (53) and test (23) set in the ratio of 7:3. A stratified-ten-fold cross-validation (CV) was conducted using the training data. 15 base predictive models were compared for performance evaluation, including CatBoost Classifier (CATBOOST), Ridge Classifier (RIDGE), Linear Discriminant Analysis (LDA), SVM-Linear Kernel (SVM), Decision Tree Classifier (DT), Random Forest Classifier (RF), Naive Bayes (NB), Logistic Regression (LR), Dummy Classifier (DUMMY), The Extra Trees Classifier (ET), Light Gradient Boosting Machine (LGBM), The Gradient Boosting Classifier (GBC), Quadratic Discriminant Analysis (QDA), K Nearest Neighbors (KNN) Classifier, Extreme Gradient Boosting (XGBOOST) [26]. The augmentation and feature engineering techniques were as follows, outliers were removed using the Singular Value Decomposition (SVD) method in the training set [27], new polynomial features were generated using existing numeric features with a degree of two [28]. Six performance metrics [26], including accuracy, precision, recall, F1 score, Matthews correlation coefficient (MCC), and area under the ROC Curve (AUC), were adopted to evaluate the discrimination of models. The top five CV scores were selected to evaluate the actual performance on the test set, and the models were ranked based on their MCC scores. We generated multiple charts to visualize the performance of the optimal model, including the AUC chart, the classification report, the confusion matrix, the SHAP value chart and the calibration plot. Brier score was calculated to evaluate the calibration. Additionally, we conducted an ablation study by using data augmentation and feature engineering. The learning algorithms were provided by PyCaret 3.3.0 (https://pycaret.org/) using Python 3.7.0.

### Ethics approval and consent to participate

This study adhered to the Declaration of Helsinki, and the study protocol was approved by the Ethics Committee of Tianjin Medical University Eye Hospital (No. 2022KY [L]-60). Informed consent was obtained from all included subjects. All methods were performed in accordance with the relevant guidelines and regulations. All patient identifiers (name, ID number, medical record number) were removed and replaced with a unique random code for data analysis and storage.

## Results

### Demographic characteristics

A total number of 76 patients were enrolled in the study, including 40 males and 36 females, with the age range 18–85 years (average 51.4 ± 15.5 years) and the median history 8 (3, 24) months. 71 cases were unilateral, 39 in the right eye and 32 in the left eye; other 5 cases were bilateral. Histopathological subtypes included: (1) Malignant tumors in 41 cases, arising from epithelium in 29 cases (19 cases of adenoid cystic carcinoma, 5 of adenocarcinoma, 2 of squamous carcinoma, one of mucinous adenocarcinoma, one of carcinoma ex-pleomorphic adenoma, and one of epithelial-myoepithelial carcinoma) and non-epithelium in 12 cases (12 cases of lymphoma); (2) Benign tumors in 35 cases, arising from epithelium in 24 cases (all of which were pleomorphic adenomas) and non-epithelium in 11 cases (benign lymphoid hyperplasia, inflammatory pseudotumor, and Mikulicz disease). No significant differences were observed in age (p=0.114), gender (p=0.790), laterality (p=0.855) and medical history (p=0.161) between the benign and malignant groups (Table 1). The test set maintains the same benign/malignant subtype distribution as the training set.

**Table 1:** Clinical and histopathologic data of 76 cases of lacrimal gland tumors.

Parameters	Total	Malignant	Benign	
	n=76 (100)	n=41 (53.9)	n=35 (46.1)	p-Value
Age ( $\overset{®}{x}$ ± *s*, year)^a^	51.4 ± 15.5	54.0 ± 14.5	48.3 ± 16.3	0.114
Male (%)^b^	40 (52.6)	21 (51.2)	19 (54.3)	0.790
Unilateral eye (%)^b^	71 (93.4)	39 (95.1)	32 (91.4)	0.855
Medical history [M (Q1, Q3), month]^c^	8 (3, 24)	9 (3, 60)	8 (2, 24)	0.161
Tissue origins^b^				
Epithelial	53 (69.7)	29 (70.7)	24 (68.6)	0.838
Non-epithelial	23 (30.2)	12 (29.3)	11 (31.4)	

^a^ *t-*test; ^b^ *χ* ^2^ *-*test; ^c^Mann–Whitney *U*‐test.

### Comparison of MRI data between benign and malignant tumors

Excellent inter and intra-reader agreements were obtained in the analysis of MRI and DCE MRI-derived parameters (Table 2). Compared with the conventional MRI features of benign and malignant tumors, there were statistically significant differences in tumor diameter (p=0.009), tumor shape (p<0.001) and adjacent bone damage (p=0.004). However, there were no statistically significant differences in T1 (p=0.724), T2 (p=0.688), and cystic changes (p=1.000). A large tumor with wedge sign and adjacent bone damage were the most significant characteristics of the malignant lacrimal tumors displayed on conventional MRI images. Clear and round posterior margins squeezing the eyeball and the adjacent bone wall were the typical characteristics of benign tumors. Contrast by the DCE-MRI parameters, K_ep_ (p=0.045) and TIC (p=0.023) were significant different between benign and malignant groups. No significant difference was observed in K^trans^ (p=0.082) and V_e_ (p=0.111). K_ep_ showed a significant higher value in malignancy. The most common TIC was type-I in benign tumors and type-II in malignant tumors.

**Table 2:** Comparison of benign and malignant tumors.

Imaging features or parameters	Malignant	Benign	p-Value	ICC	
	n=41 (53.9)	n=35 (46.1)		Inter	Intra
Tumor diameter ( $\overset{®}{x}$ ± *s*, cm)^a^	3.0 ± 1.0	2.4 ± 0.8	0.009	0.978	0.956
Tumor shape (%)^b^					
Oval	9 (21.9)	23 (65.7)	0.000	0.955	0.924
Wedge sign	20 (48.8)	10 (28.6)			
Other	12 (29.3)	2 (5.7)			
Cystic change (%)^b^					
Without	4 (9.8)	3 (8.6)	1.000	1.000	1.000
With	37 (90.2)	32 (91.4)			
Adjacent bone damage (%)^b^					
Without	24 (58.5)	31 (88.6)	0.004	1.000	1.000
With	17 (41.5)	4 (11.4)			
T1 signal intensity (%)^b^					
Hypointense	0 (0)	0 (0)			
Isointense	38 (92.7)	34 (97.1)	0.724	1.000	1.000
Hyperintense	3 (7.3)	1 (2.9)			
T2 signal intensity (%)^b^					
Hypointense	2 (4.9)	3 (8.6)	0.688	0.911	0.925
Isointense	23 (56.1)	21 (60.0)			
Hyperintense	16 (39.0)	11 (31.4)			
Enhancement homogeneity (%)^b^					
Homogeneous	24 (58.5)	29 (82.9)	0.021	0.937	0.911
Inhomogeneous	17 (41.5)	6 (17.1)			
K^trans^ [M (Q_1_, Q_3_), min^−1^]^c^	0.213 (0.171, 0.254)	0.159 (0.133, 0.256)	0.082	0.985	0.965
K_ep_ [M (Q_1_, Q_3_), min^−1^]^c^	0.389 (0.278, 0.501)	0.288 (0.224, 0.467)	0.045	0.990	0.986
V_e_ ( $\overset{®}{x}$ ± *s*)^a^	0.594 ± 0.180	0.656 ± 0.154	0.111	0.958	0.923
TIC (%)^b^					
I	13 (31.7)	22 (62.9)	0.023	0.974	0.974
II	17 (41.5)	7 (20.0)			
Ⅲ	11 (26.8)	6 (17.1)			

^a^ *t-*test; ^b^ *χ* ^2^ *-*test; ^c^Mann–Whitney *U*‐test. ICC, intraclass correlation coefficient.

### Predictive models assisted diagnosis of benign and malignant tumors

**Figure 1:**
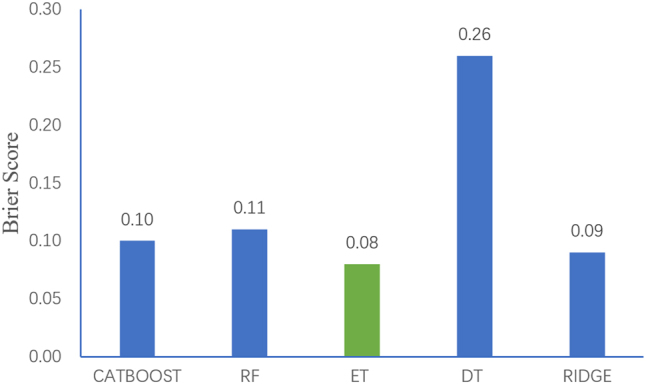
Brier score of the five top model for tumor type task (conventional MRI+DCE-MRI).

The selective parameters at a significance level of p<0.15 were entered into predictive models. Predictive models with conventional MRI features (Table 3) and predictive models with DCE quantitative parameters plus conventional MRI features (Table 4) were conducted separately to compare the diagnostic value. The six-performance metrics of models with DCE quantitative parameters was significantly better than the models without. Based on the CV results in table 4, the top five models were Catboost, RF, ET, DT and Ridge. The top model was Catboost, with an MCC score of 0.62 and F1 score of 0.81. However, the test scores presented a little different ranking among the five, where ET showed the highest MCC (0.84) and the highest F1 score (0.91), as well as the accuracy (0.91), precision (1.00) and AUC (0.95). The inconsistent model ranking between CV and test scores showed that other models were overfitting the data, while ET was possibly under-trained. Besides, ET model showed the lowest brier score (0.08), therefore, the ET was selected as the final model (Figure 1).

**Table 3:** Model performance for the tumor type task (conventional MRI).

		Accuracy	AUC	Recall	Prec	F_1_	MCC
CV	NB	0.68	0.77	0.52	0.87	0.61	0.43
	RIDGE	0.67	0.72	0.67	0.76	0.66	0.41
	ET	0.70	0.77	0.73	0.80	0.73	0.40
	DT	0.69	0.70	0.67	0.76	0.68	0.40
	RF	0.69	0.71	0.67	0.76	0.68	0.40

Test	NB	0.61	0.70	0.42	0.71	0.53	0.26
	RIDGE	0.61	0.60	0.75	0.60	0.67	0.21
	ET	0.65	0.63	0.75	0.64	0.69	0.30
	DT	0.61	0.58	0.75	0.60	0.67	0.21
	RF	0.61	0.71	0.75	0.60	0.67	0.21

Conventional MRI included tumor shape, tumor diameter and bone damage. Acc, accuracy; AUC, area under the ROC curve; Prec, precision; MCC, Matthews correlation coefficient; CV, cross-validation; NB, Naive Bayes; ET, extra trees classifier; RIDGE, ridge classifier; RF, random forest classifier; LR, logistic regression.

**Table 4:** Model performance for the tumor type task (conventional MRI+DCE-MRI).

		Accuracy	AUC	Recall	Prec	F_1_	MCC
CV	CATBOOST	0.80	0.85	0.83	0.82	0.81	0.62
	RF	0.78	0.81	0.83	0.80	0.80	0.60
	ET	0.78	0.86	0.80	0.82	0.78	0.60
	DT	0.78	0.78	0.80	0.81	0.80	0.58
	RIDGE	0.74	0.74	0.77	0.79	0.73	0.54

Test	CATBOOST	0.87	0.93	0.83	0.91	0.87	0.74
	RF	0.83	0.92	0.83	0.83	0.83	0.65
	ET	0.91	0.95	0.83	1.00	0.91	0.84
	DT	0.74	0.73	0.83	0.71	0.77	0.47
	RIDGE	0.91	0.92	0.83	1.00	0.91	0.83

Conventional MRI included tumor shape, tumor diameter and bone damage; DCE-MRI included TIC, K^trans^, K_ep_, V_e_, TIC, time-intensity curve; ET, extra trees classifier; CATBOOST, catboost classifier; DT, decision tree classifier; RF, random forest classifier; ET, extra trees classifier; KNN, K nearest neighbors.

### The model performance of ET based on DCE quantitative parameters plus conventional MRI features

**Figure 2:**
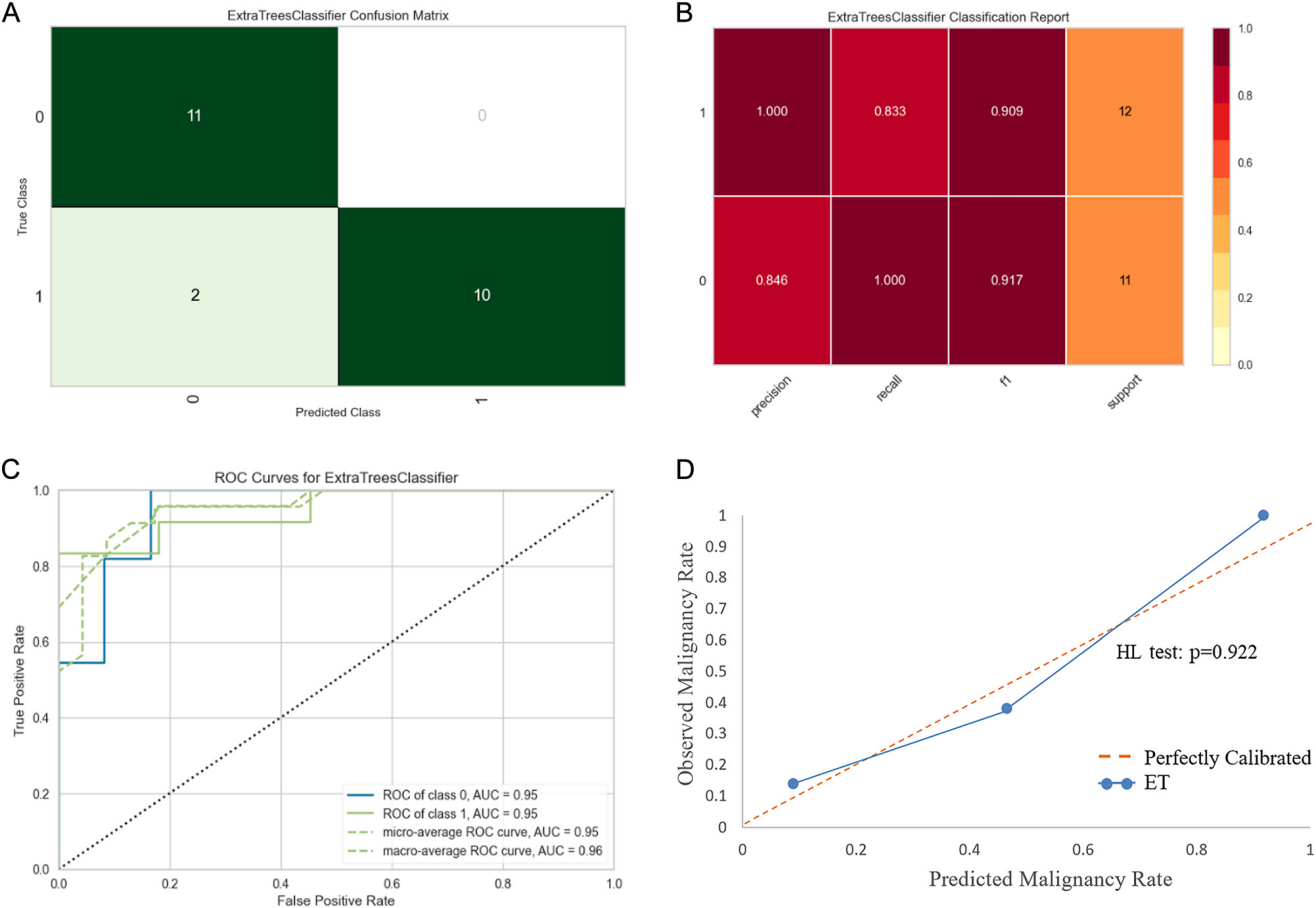
Performance charts for the best model (ET) of the tumor type task. (a) Confusion matrix; (b) classification report; (c) ROC curve; (d) calibration plot.

**Figure 3:**
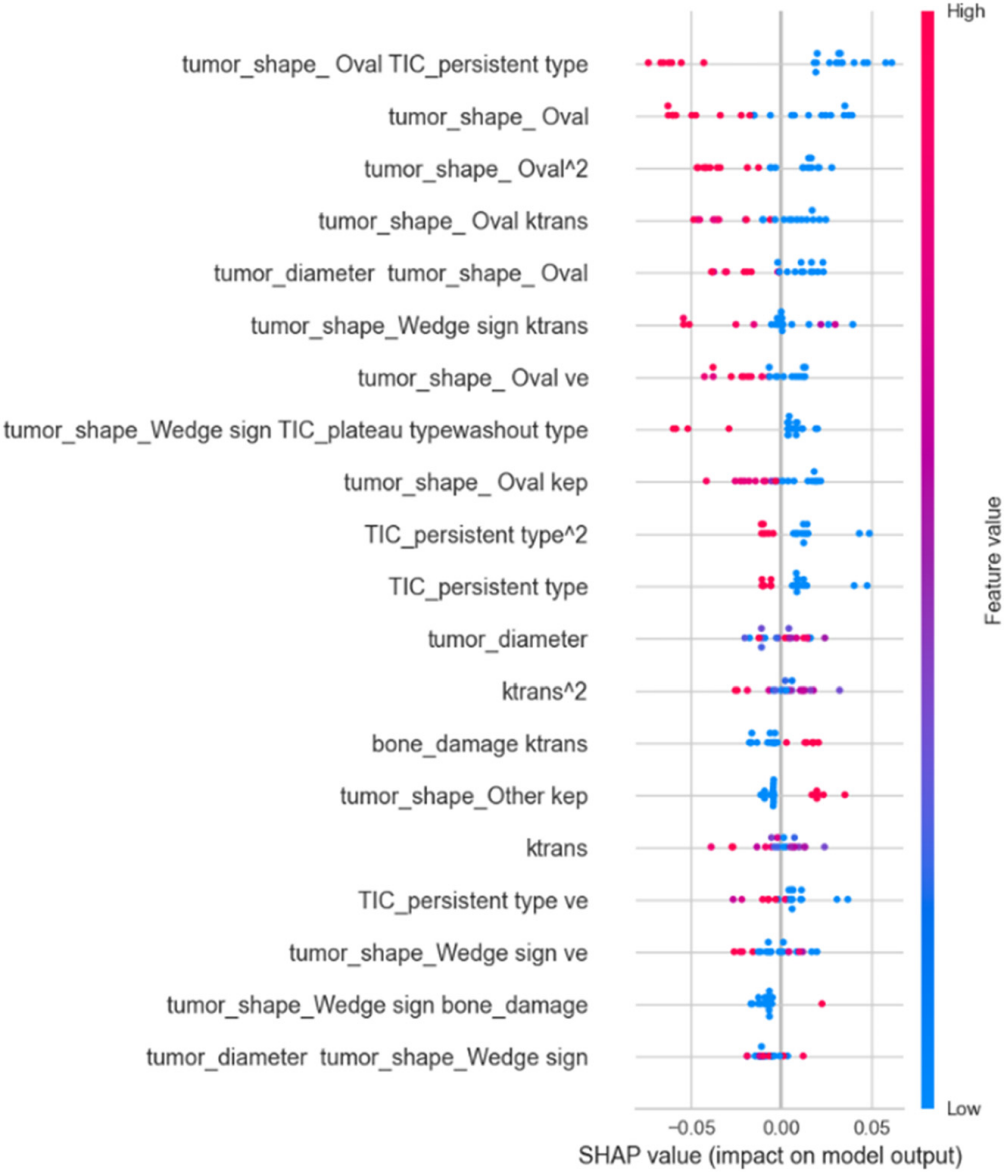
SHAP value chart for the best model (ET) of the tumor type task. Positive SHAP values indicate an increased risk of malignancy.

Figure 2 showed four subfigures of the ET’s performance, including confusion matrix, classification report, ROC curve and calibration plot. The confusion matrix shows the exact numbers of the four outcomes, including true positive (TP), true negative (TN), false positive (FP), and false negative (FN), it was noted that two positive (malignant) samples were misclassified as negative (benign), and no negative samples were misclassified as positive. The classification report showed precision, recall, F1, and support for each class, where support suggested the number of samples for a class. For this task, the F1 scores showed high and similar value (0.909 vs. 0.917) between the two classes, which indicated a good performance for both positive and negative classes. Since the classes were more balanced, the micro- and macro-average AUCs were similar (0.95 vs. 0.96). Calibration plot showed a good fit between perfectly calibrated and ET prediction with the p value of 0.922 in Hosmer-Lemeshow test. Lastly, the SHAP value chart ranked interaction between not oval tumor shape nor TIC persistent type, not oval tumor shape, interaction between not oval tumor shape and high K^trans^ value, were the top predictive factors which suggesting an increased risk of malignancy (Figure 3).

### Data augmentation and feature engineering

**Figure 4:**
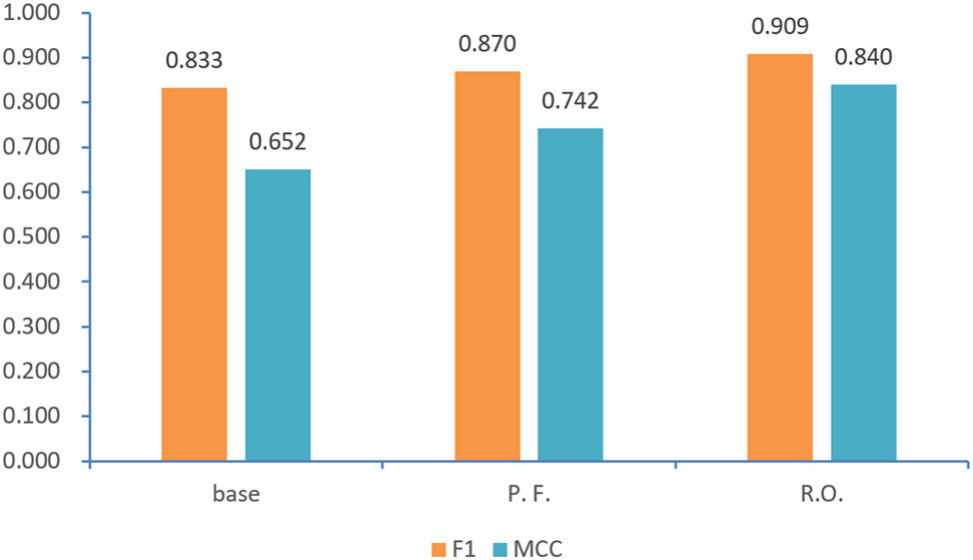
The ablation study demonstrated a gradual increase in performance of the baseline model with each data augmentation and feature engineering method employed. Abbreviations include R.O. (remove outlier), P.F. (polynomial feature). The sequence of data augmentation and feature engineering: baseline → remove outliers → add polynomial features.

We conducted an ablation study to assess the individual contributions of these components towards the overall model by incrementally adding each data augmentation/feature engineering method onto the baseline model. The results are displayed in Figure 4. The highest F1 has been lifted from 0.833 to 0.909, and the highest MCC has been lifted from 0.652 to 0.840. It can be observed that the F1/MCC score has consistently improved for the models from left to right, as each data augmentation/feature engineering method is added. The uptrend indicates the positive effects of these methods.

## Discussion

In the present study, we found that predictive models combining DCE quantitative parameters and conventional MRI features outperformed those based solely on conventional MRI features. Among these models, ET demonstrated superior performance in diagnosing benign and malignant lacrimal tumors.

Conventional MRI can provide specific features of adjacent bone damage and tumor shape for malignant lacrimal tumors. The diagnosis of bone damage in MRI relies on the margin characteristics of the tumor and morphological changes in the adjacent bone [29]. Williams et al. [30] reported that almost all lacrimal gland adenoid cystic carcinoma with a diameter larger than 2.0 cm had periosteum or bone invasion established by pathologic examination, and the sensitivity of the imaging examination to bone erosion was 78.6 %. In this study, it presented a low sensitivity of MRI diagnosis for bone damage, with cases of adjacent bone damage accounting for 41.5 % of malignant tumors. This could be attributed to the predominant manifestation of compressive changes in the adjacent orbital bone by partial malignant tumors (lymphoma). A more informative MRI feature compared to bone damage was the tumor’s posterior margin characteristic. Specifically, we found 78.1 % of malignant tumors exhibited either a wedge sign (48.8 %) or an irregular shape (29.3 %) at the posterior margin. Young et al. [23] reported that wedge sign was more likely the imaging manifestation of malignant lacrimal tumor growing posteriorly along the orbital roof or lateral wall, which was due to a restriction of the tumor growth by the intermuscular septum and Tenon’s sheath around the neighboring recti. Moreover, conventional MRI transverse section, particularly the T2WI fat-suppression sequence, can effectively visualize the tumor’s posterior margin and ensure the continuity of the cortical bone contour. It is important to note that the typical features of conventional MRI may be influenced in cases where the benign tumor extends to the orbital apex and is of significant size, or when a malignant tumor exhibits small size and round shape. Therefore, additional imaging modalities should be employed to enhance diagnostic sensitivity.

The value of DCE-MRI in the diagnosis of benign and malignant lacrimal tumors deserves to be further explored. It is widely reported that TIC types have certain diagnostic value in benign and malignant orbital tumors, such as type-II (plateau type) or type-III (washout type) curve is the characteristic enhancement mode of malignant tumors [17], 31]. However, TIC types are subjective data and cannot provide underlying pharmacokinetic information in tissue [32]. Studies have shown that it’s often necessary to combine with multiple quantitative parameters to improve the results. Wang et al. [21] reported the diagnostic specificity of single quantitative parameter is not high, and the combination of volume transfer constant (K^trans^) and apparent diffusion coefficient (ADC) presented a predictive sensitivity of 93.3 %, specificity of 87.2 %, and accuracy of 89.6 %, while Li et al. [20] reported a similar predictive accuracy of 86.1 % by combination TIC and ADC. Unlike previous studies that primarily relied on conventional logistic regression models, our study employed 15 base predictive models, the linear and non-linear ensemble method capable of capturing complex interactions between DCE-MRI quantitative parameters and conventional MRI morphological features. The ET model based on DCE quantitative parameters combined with conventional MRI features achieved the accuracy of 91 %, precision of 100 % and recall (sensitivity) rate of 83 %, which highlight its superior performance with prior diagnostic models for lacrimal gland tumors. Consequently, the integration of machine learning algorithms and model-dependent MRI diagnosis represents a pivotal area for further investigation.

The models of machine learning algorithms have been used after training to predict the two-state label [33]. Models based on “trees” are generally better than those based on linear squares logistic models. The ET model, which applies several randomized Decision Tree Classifier (DT) to different subsamples of the dataset, is selected as the final model from 15 base predictive models. The DTs are subsequently employed for data classification, offering advantages such as the utilization of randomized DTs for subsamples and mitigated overfitting through result averaging. Therefore, they are better than one single DT [34]. Evaluation for models in machine learning commonly includes metrics such as accuracy, precision, recall, F1, MCC, and AUC. The F1 score represents the harmonic mean of accuracy and recall, while MCC is a robust statistical measure that treats true class and predicted class as binary variables to calculate their correlation coefficients. The stronger the correlation between the true value and the predicted value, the more accurate the prediction becomes. It yields a high score only if the prediction achieves favorable outcomes across all four categories of confusion matrix [35]. The results demonstrate that ET achieves the highest MCC and F1 values in the test dataset, as well as exhibiting excellent calibration with predicted risk closely aligned to observed risk. These findings indicate an effective lacrimal gland tumor classification model by incorporating multiple MRI parameters.

The DCE quantitative parameters, that reflect tissue perfusion or permeability, are closely related to the tumor’s microvascular environment. Therefore, the heterogeneity of their subjects (intraocular and orbital, benign and malignant, epithelial and non-epithelial tumors) could result in mixed conclusions between studies. Ro et al. [16] demonstrated that the higher values of K^trans^ and K_ep_ suggested the potential presence of a malignant tumor, whereas in the other two investigations, K_ep_ and V_e_ had significant diagnostic performance [36], 37]. There is similar pathological and image complexity in lacrimal gland tumors, and more studies should be included to validate their diagnostic value. The SHAP value in our findings have been used to rank the MRI parameters based on their importance, thereby indicating their significant impact on the model output. Notably, not oval tumor shape, nor TIC persistent type, and high K^trans^ value have emerged as significant predictive parameters, which contribute most to the model’s predictions. Consequently, DCE serves as a valuable adjunct to conventional MRI, facilitating the identification of sensitive features through ET modeling for accurate prediction of lacrimal gland tumors.

The incidence of lacrimal gland tumors is relatively low, and the integration of DCE-MRI and machine learning models into imaging diagnosis may face practical challenges. The small single-center sample size may limit generalizability, though stratified cross-validation and robust performance metrics mitigate overfitting risk. The study also lacks external validation, which is crucial for assessing the clinical utility of the predictive models. Future studies should aim to include a larger and multi-center studies to validate the results. Additionally, the extraction of DCE parameters relies on manually delineated ROI and a single pharmacokinetic model. While this approach offers convenience for clinical application, it may introduce bias in data selection. Future research will focus on implementing deep learning-based segmentation models and developing semi-automatic or automatic ROI delineation methods. Additionally, the feasibility of integrating the ET model into clinical workflow as a visualization tool (e.g., a radiomics workstation plug-in) warrants further investigation.

This study explores the effective lacrimal gland tumor classification models by incorporating multiple MRI parameters. The predictive models based on DCE quantitative parameters plus conventional MRI features exhibit superior performance compared to those based solely on conventional MRI features. Among these models, ET can be considered as the optimal model in the diagnosis of benign and malignant lacrimal tumors.

## References

[1] Shields CL, Shields JA, Eagle RC, Rathmell JP (1989). Clinicopathologic review of 142 cases of lacrimal gland lesions. Ophthalmol.

[2] von Holstein SL, Therkildsen MH, Prause JU, Stenman G, Siersma VD, Heegaard S (2013). Lacrimal gland lesions in Denmark between 1974 and 2007. Acta Ophthalmol.

[3] von Holstein SL, Coupland SE, Briscoe D, Le Tourneau C, Heegaard S (2013). Epithelial tumours of the lacrimal gland: a clinical, histopathological, surgical and oncological survey. Acta Ophthalmol.

[4] Bernardini FP, Devoto MH, Croxatto JO (2008). Epithelial tumors of the lacrimal gland: an update. Curr Opin Ophthalmol.

[5] Woo KI, Yeom A, Esmaeli B (2016). Management of lacrimal gland carcinoma: lessons from the literature in the past 40 years. Ophthalmic Plast Reconstr Surg.

[6] Garner A (1992). Orbital lymphoproliferative disorders. Br J Ophthalmol.

[7] Wang XN, Qian J, Yuan YF, Zhang R, Zhang YQ (2017). Application of rose and Wright’s algorithm in the diagnosis of lacrimal gland masses: a study of 93 cases. Can J Ophthalmol.

[8] Ben Simon GJ, Annunziata CC, Fink J, Villablanca P, McCann JD, Goldberg RA (2005). Rethinking orbital imaging establishing guidelines for interpreting orbital imaging studies and evaluating their predictive value in patients with orbital tumors. Ophthalmology.

[9] Gaddikeri S, Gaddikeri RS, Tailor T, Anzai Y (2016). Dynamic contrast-enhanced MR imaging in head and neck cancer: techniques and clinical applications. AJNR Am J Neuroradiol.

[10] Ota Y, Liao E, Capizzano AA, Kurokawa R, Bapuraj JR, Syed F (2021). Diagnostic role of diffusion-weighted and dynamic contrast-enhanced perfusion MR imaging in paragangliomas and schwannomas in the head and neck. AJNR Am J Neuroradiol.

[11] Ota Y, Liao E, Capizzano AA, Yokota H, Baba A, Kurokawa R (2022). MR diffusion and dynamic-contrast enhanced imaging to distinguish meningioma, paraganglioma, and schwannoma in the cerebellopontine angle and jugular foramen. J Neuroimaging.

[12] Ewing JR, Bagher-Ebadian H (2013). Model selection in measures of vascular parameters using dynamic contrast-enhanced MRI: experimental and clinical applications. NMR Biomed.

[13] Park H, Kim SH, Kim JY (2022). Dynamic contrast-enhanced magnetic resonance imaging for risk stratification in patients with prostate cancer. Quant Imag Med Surg.

[14] Wei W, Jia G, von Tengg-Kobligk H, Heverhagen JT, Abdel-Rahman M, Wei L (2017). Dynamic contrast-enhanced magnetic resonance imaging of ocular melanoma as a tool to predict metastatic potential. J Comput Assist Tomogr.

[15] Petralia G, Summers PE, Agostini A, Ambrosini R, Cianci R, Cristel G (2020). Dynamic contrast-enhanced MRI in oncology: how we do it. Radiol Med.

[16] Ro SR, Asbach P, Siebert E, Bertelmann E, Hamm B, Erb-Eigner K (2016). Characterization of orbital masses by multiparametric MRI. Eur J Radiol.

[17] Yuan Y, Kuai XP, Chen XS, Tao XF (2013). Assessment of dynamic contrast-enhanced magnetic resonance imaging in the differentiation of malignant from benign orbital masses. Eur J Radiol.

[18] Jittapiromsak N, Hou P, Liu HL, Sun J, Schiffman JS, Chi TL (2018). Dynamic contrast-enhanced MRI of orbital and anterior visual pathway lesions. Magn Reson Imaging.

[19] Ang T, Juniat V, Patel S, Selva D (2024). Evaluation of orbital lesions with DCE-MRI: a literature review. Orbit.

[20] Li XF, Wu X, Qian J, Yuan YF, Wang SJ, Ye XP (2022). Differentiation of lacrimal gland tumors using the multi-model MRI: classification and regression tree (CART)-based analysis. Acta Radiol.

[21] Wang Y, Song L, Guo J, Xian J (2020). Value of quantitative multiparametric MRI in differentiating pleomorphic adenomas from malignant epithelial tumors in lacrimal gland. Neuroradiology.

[22] Jian T, Yang M, Wu T, Ji X, Xia S, Sun F (2024). Diagnostic value of dynamic contrast enhancement combined with conventional MRI in differentiating benign and malignant lacrimal gland epithelial tumours. Clin Radiol.

[23] Young SM, Kim YD, Shin HJ, Imagawa Y, Lang SS, Woo KI (2019). Lacrimal gland pleomorphic adenoma and malignant epithelial tumours: clinical and imaging differences. Br J Ophthalmol.

[24] Lorenzano D, Rose GE (2017). The “Wedge Sign”: an imaging sign for aggressive lacrimal gland disease. Ophthalmology.

[25] Gunduz K, Shields CL, Gunalp I, Shields JA (2003). Magnetic resonance imaging of unilateral lacrimal gland lesions. Graefes Arch Clin Exp Ophthalmol.

[26] Gao F, Xiao Z, Chen S, Yu R, Li X (2024). MedGCN: an IoT-edge thrombus graph convolutional network for accurate prediction and prescription diagnosis of vascular occlusive diseases from unstructured clinical reports. Comput Commun.

[27] Khoshrou A, Pauwels EJ Data-Driven Pattern Identification and Outlier Detection in Time Series2019.

[28] Zheng A, Casari A (2018). Feature engineering for machine learning: principles and techniques for data scientists.

[29] Gamoh S, Akiyama H, Tsuji K, Nakazawa T, Morita S, Tanaka A (2018). Non-contrast computed tomography and magnetic resonance imaging features of mucoepidermoid carcinoma in the salivary glands. Oral Radiol.

[30] Williams MD, Al-Zubidi N, Debnam JM, Shinder R, DeMonte F, Esmaeli B (2010). Bone invasion by adenoid cystic carcinoma of the lacrimal gland: preoperative imaging assessment and surgical considerations. Ophthalmic Plast Reconstr Surg.

[31] Zheng N, Li R, Liu W, Shao S, Jiang S (2018). The diagnostic value of combining conventional, diffusion-weighted imaging and dynamic contrast-enhanced MRI for salivary gland tumors. Br J Radiol.

[32] Chikui T, Obara M, Simonetti AW, Ohga M, Koga S, Kawano S (2012). The principal of dynamic contrast enhanced MRI, the method of pharmacokinetic analysis, and its application in the head and neck region. Int J Dent.

[33] Li Y, Huang C, Ding L, Li Z, Pan Y, Gao X (2019). Deep learning in bioinformatics: introduction, application, and perspective in the big data era. Methods.

[34] Shalev-Shwartz S, Ben-David S (2014). Understanding machine learning: from theory to algorithms.

[35] Chicco D, Jurman G (2020). The advantages of the matthews correlation coefficient (MCC) over F1 score and accuracy in binary classification evaluation. BMC Genom.

[36] Hu H, Xu XQ, Liu H, Hong XN, Shi HB, Wu FY (2017). Orbital benign and malignant lymphoproliferative disorders: differentiation using semi-quantitative and quantitative analysis of dynamic contrast-enhanced magnetic resonance imaging. Eur J Radiol.

[37] Xu XQ, Qian W, Ma G, Hu H, Su GY, Liu H (2017). Combined diffusion-weighted imaging and dynamic contrast-enhanced MRI for differentiating radiologically indeterminate malignant from benign orbital masses. Clin Radiol.

